# Asthma pressurised metered dose inhaler performance: propellant effect studies in delivery systems

**DOI:** 10.1186/s13223-017-0202-0

**Published:** 2017-06-29

**Authors:** William F. S. Sellers

**Affiliations:** Broadgate House, 22 Broadgate, Great Easton, Leicestershire, LE16 8SH UK

**Keywords:** Metered dose inhalers, Propellants, Valved holding chambers (spacers), Ventilator inhaler devices, Toxicology

## Abstract

**Background:**

Current pressurised metered dose asthma inhaler (pMDI) propellants are not inert pharmacologically as were previous chlorofluorocarbons, have smooth muscle relaxant‚ partial pressure effects in the lungs and inhaled hydrofluoroalkane 134a (norflurane) has anaesthetic effects. Volumes of propellant gas per actuation have never been measured.

**Methods:**

In-vitro studies measured gas volumes produced by pMDIs on air oxygen (O_2_) levels in valved holding chambers (VHC) and the falls in O_2_% following actuation into lung ventilator delivery devices.

**Results:**

Volumes of propellant gas hydrofluoroalkane (HFA) 134a and 227ea and redundant chlorofluorocarbons (CFC) varied from 7 ml per actuation from a small salbutamol HFA inhaler to 16 ml from the larger. Similar-sized CFC pMDI volumes were 15.6 and 20.4 ml. Each HFA salbutamol inhaler has 220 full volume discharges; total volume of gas from a small 134a pMDI was 1640 ml, and large 3885 ml. Sensing the presence of liquid propellant by shaking was felt at the 220th discharge in both large and small inhalers. Because of a partial pressure effect, VHC O_2_% in air was reduced to 11% in the smallest 127 ml volume VHC following 10 actuations of a large 134a salbutamol inhaler. The four ventilator delivery devices studied lowered 100% oxygen levels to a range of 93 to 81% after five actuations, depending on the device and type of pMDI used.

**Conclusion:**

Pressurised inhaler propellants require further study to assess smooth muscle relaxing properties.

## Background

Inhaled therapy delivery devices for asthma and chronic lung disease such as oxygen driven jet nebulisers, pressurised metered dose inhalers (pMDI), and valved holding chambers (VHC) have little evidence or research to support their efficacy [1, 2]; historical use determines current practice. The amounts of drugs deposited, and in which part of the respiratory tract absorption occurs, is based on intuition rather than research. Inhaled asthma drugs are erroneously considered to act directly through local absorption into receptors, and not systemically. The physical and pharmacological properties of the propellant and drugs in pMDIs gives a scientific explanation and reason for the increasing use of pMDIs and VHCs to manage acute severe asthma in place of evidence-baseless jet nebulisation of beta2-agonists in saline. Inhaled anaesthetic agents and stupefants (glue, butane) descend deep into the lung architecture where bronchial and alveolar absorption into blood is followed by vascular delivery to the whole body. Beta2-agonists in respiratory disease arrive at bronchial smooth muscle receptors from bronchial arteries, and one cardiac cycle later by pulmonary arteries. Inhaled terbutaline has been studied for uterine tocolysis. Upper respiratory tract and oral absorption also play an unknown-percentage part in the delivery of inhaled drugs to target organs. There are corticosteroid receptors in the bronchial epithelial lining cells, which may account for the thought that inhaled drugs act locally and remain in the lungs. The propellants of pMDIs have had little scrutiny, either of previous chlorofluorocarbon (CFC) di-chlorodi-fluoro and tri-chlorofluoro methanes, current hydrofluoroalkanes (HFA) 134a and 227ea. HFA (hydrofluorocarbon) inhaler propellants replaced chlorofluorocarbons in the late 1990s, but propellant toxicological research was incomplete and an anaesthetic effect of HFA134a was missed. Asthma inhaler actuation produces a measurable volume of propellant gases, CFCs, HFA134a, HFA227ea, and as per John Dalton’s Law of partial pressures, oxygen and nitrogen in air are reduced in the lungs during inhalation. Inhaler abuse for recreational purposes occurs and Olympic endurance athletes with asthma have out-performed their healthy rivals since the year 2000 [3, 4], interestingly mirroring replacement of inert CFCs by the improved delivery effects of pharmacologically active HFA propellants.

Volumes of propellant produced by metered dose inhalers have not before been measured, nor the volumes of valved holding chambers (VHC) or the gas (percentage) changes inside them after actuations of pMDIs. Devices specifically made to be inserted into ventilated patient circuits for actuation of aluminium pMDI cartridges have received little scientific appraisal. Toxicology studies on safety for human use of HFA134a and 227ea neither appreciated nor studied the physical and pharmacological properties of the propellant at higher doses than 0.8% in humans [5, 6]. HFA134a and 227ea are gases at room temperature. All fluorinated hydrocarbon inhalational anaesthetic agents have smooth muscle relaxing properties in the gut, vasculature, uterus, and lungs (bronchial smooth muscle) via a calcium channel blocking effect, which is the mechanism of action of magnesium sulphate and beta2-agonists. Both HFA propellants interfered with infra-red anaesthetic agent operating theatre monitors [7]. In a review it was noted that up to twelve actuations of metered dose inhalers into valved holding chambers before a single inhalation were being used to treat acute severe asthma in children’s hospitals in Melbourne and Sydney, Australia and there was a similar use by United Kingdom paediatricians [2]. A closer examination of metered dose inhaler publications revealed a lack of information of the pharmacological and physical effects of current propellants. This review looks at the performance of pressurised metered dose inhalers in vitro and discusses toxicology studies performed to assess propellant safety and publications comparing CFC and HFA inhalers. Some of the results presented have been published as abstracts [8, 9]. Simple measurements in vitro were made of CFC and HFA pMDIs, valved holding chambers, and tracheal delivery pMDI apparatus [10].

## Methods

In vitro experiments were performed using anaesthetic equipment and oxygen monitors; pMDIs were well shaken prior to actuation.

### Actuation volumes of inhalers

**Fig. 1:**
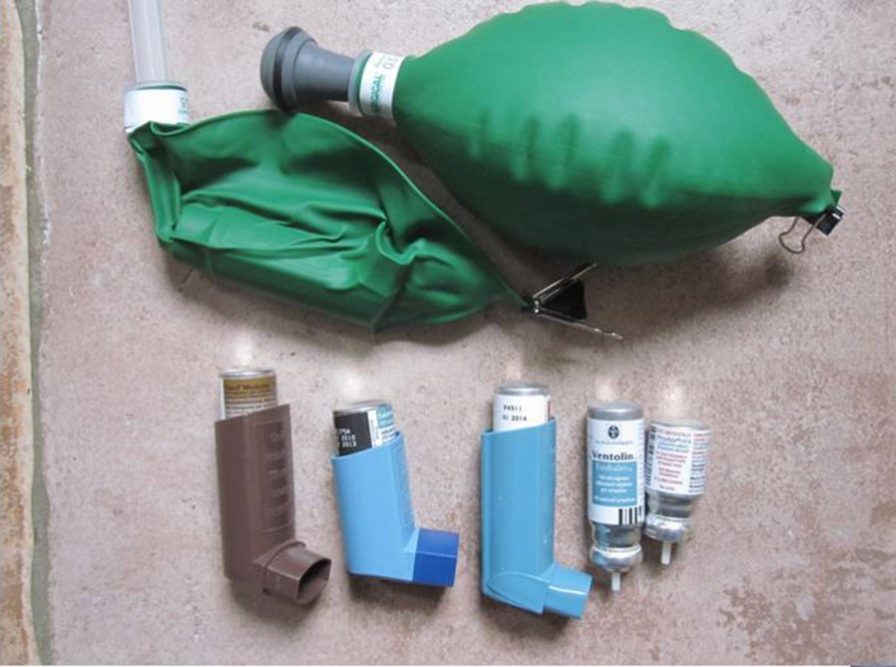
Full and evacuated 500 ml anaesthetic reservoir bags. The evacuated contains an inhaler and other inhalers and cartridges are shown

HFA134a propelled salbutamol small aluminium canister (Salamol^R^, IVAX Pharmaceuticals, Waterford, Ireland), salbutamol large aluminium canister (Ventolin™, Allen & Hanburys, Uxbridge, UK) inhalers and a large Clenil^R^Modulite^R^ (Chiesi, Cheadle, UK) beclometasone corticosteroid steroid inhaler containing 13% ethanol (which aids corticosteroid solubility); HFA227ea propelled sodium cromogliate (Intal™, SanofiAventis, Guildford, Surrey, UK) and budesonide/eformoterol (Vannair™ 100/6, AstraZeneca, Auckland, NZ), CFC (trichlorofluoromethane and dichlorodi-fluoromethane) propelled small canister salmeterol (Serevent™), expiration date 2005, and large salbutamol (Ventolin™), expiration 1995 (both Allen & Hanburys), were placed inside 500 ml green reservoir bags of an anaesthetic circuit (Intersurgical, Wokingham, UK). The bags were sealed and evacuated to empty, and shaken inhaler actuations by hand, from outside the reservoir bag, were counted until the bag was deemed full (Fig. 1). This was repeated three times for each inhaler, the bag was evacuated using a 60 ml syringe until empty. The volume in the bag was divided by the number of actuations to give a volume in millilitre (ml) per actuation or “puff”.

### Weights, total volumes and volumes toward exhaustion of pMDI propellant

Small and large salbutamol pMDI full and empty aluminium cannisters were weighed on hospital biochemistry laboratory scales. Total volumes were measured until exhaustion of the inhaler by reservoir bag insertion, as above. Volumes were measured after every 50 actuations. At 150 and up to 200, 210, 220 and 230 discharges, the pMDI was removed from the reservoir bag and propellant liquid movement was “sensed” by both shaking and listening within close earshot. After 200 discharges, each 10 actuations were measured until exhaustion. Using Avogadro’s Hypothesis or Law (Gram molecular weight = 22.4 l) but without subtracting salbutamol powder weight (>20mg) and the 1% weight by volume of ethanol, theoretical volumes of each net weight of propellant were found.

### Actuations into valved holding chambers (spacers)

Four types of VHCs, recommended in order to reduce the velocity of the expelled propellant, to improve timing of inhalation and to warm the propellant and drug [11] were studied. A valve at the inspiratory end retains gas and drug in the chamber reducing leakage to atmosphere before inhalation. A large salbutamol Ventolin^R^ pMDI was activated into an Ablespacer™ (Clement Clarke International, Essex, UK), Vortex^R^ (Pari, VA, USA), BabyHaler^R^ (GlaxoSmithKline, Evreux, France) and Volumatic™ (Allen and Hanbury, Middlesex, UK). The volumes of the spacers (which were unknown), were determined by filling them with water, emptying and collecting the water, weighing the water and assuming 1 g of water represents 1 ml volume. Oxygen falls were measured after one, two and ten actuations of a large salbutamol pMDI into the four spacers using oxygen and CO_2_ tubing inserted into the spacers, which delivered the gases to a Datex paramagnetic oxygen analyser (measuring gas as a percentage) sampling at 150 ml/min. Spacers have inspiratory valves which help reduce leak of propellant and drug from the device; on a deep inhalation air will be entrained though the pMDI. VHCs are “leaky” and addition of pressurised gas from pMDIs displaces existing air and pressure remains atmospheric. The Vortex VHC has an aluminium interior sleeve which is said to reduce a static effect which can cause adherence of drug to the walls of pure plastic spacers.

### Oxygen falls in a ventilator circuit reservoir bag using four pMDI delivery devices

An anaesthetic ventilator and circle absorber tubing delivered 100% oxygen with a tidal volume of 500 ml at a rate of 12 breaths per minute in turn through each pMDI delivery device attached to a size 7 mm id 28 cm length cuffed tracheal tube inserted and sealed by tracheal cuff inflation in the opening of a 1 l anaesthetic reservoir bag. From the distal end of the bag gas contents were sampled by a Philips MP70 G5-O_2_ analyser. When 100% oxygen content of the reservoir bag was seen, shaken inhalers of norflurane propelled ipratropium and levalbuterol (Duolin™, Rex Medical Ltd, Auckland, NZ), salbutamol (Respigen™, Mylan, Auckland, NZ) and apaflurane propelled sodium cromoglycate (Intal™, SanofiAventis, Surrey, UK) and budesonide and eformoterol (Vannair™, AstraZeneca, Auckland, NZ) were separately actuated five times during the short period between ventilations into the devices. Puff volumes were determined as in Method 2. The DDS Spirale^R^ (Armstrong Medical, Coleraine, NI, UK) system was used in “open” position when it has a volume of 133 ml. It has no leak from the inhaler port when in “closed” position. The following three devices have “caps” that seal the inhaler port when not being used; an MDI delivery connector, (1964001, Intersurgical, Berkshire, UK), a single swivel tube inhaler, L-Trace (60-60-009, Jackson Allison, Auckland, NZ) and a “Three in one respiratory care system” RT 200 (Fisher & Paykel Healthcare, Auckland, NZ) which has a port as part of the distal “Y” of the tubing. The fall in oxygen percentages was recorded for each inhaler and device.

## Results

### Volumes after actuations of different inhalers

Table 1—A small salbutamol 134a pMDI produced 7 ml per actuation, larger 16 ml per puff. Larger corticosteroid produced 11.75 ml and Intal^R^ produced 13.5 ml. A small salmeterol CFC pMDI produced 15.6 ml and large salbutamol 20.4 ml per actuation. Table 1 shows volumes of propellant and drug of different pMDIs that were actuated to fill the reservoir bag. The reservoir bag cooled as the low boiling point propellant was expelled into the bag; with a large salbutamol HFA134a inhaler, the temperature fall was from 20 °C room temperature to minus 6 °C, measured with a meat thermometer, and caused little change in volume.

**Table 1:** Propellant volumes from different pMDIs

HFA 134a norflurane	Salamol, (IVAX) small	490/70 puffs = 7 ml per puff
	Ventolin (A&H) large	480/30 puffs = 16 ml per puff
	Clenil Modulite™ (Chiesi) large	400/35 puffs = 11.75 ml per puff
HFA 227ea apaflurane	Intal™ (Sanofi-Aventis) large	465/35 puffs = 13.5 ml per puff
	Vannair™ (AstraZeneca) small	480/53 puffs = 9 ml per puff
CFC^a^	Serevent™ (A&H) small	530 ml/35 puffs = 15.6 ml per puff
	Ventolin ™ (A&H) large	490/24 puffs = 20.4 ml per puff

^a^CFCs dichlorodifluoromethane Cl_2_F_2_C; trichlorofluoromethane Cl_3_FC plus lecithin

### Total weights and volumes

Table 2—220 full volume HFA propellant doses are available, after this, no liquid is sensed on shaking small or large aluminium cartridges, when removed from their plastic surrounds.

**Table 2:** Weights and volumes of salbutamol HFA134a propellant pMDIs

	Small salbutamol Salamol^R^	Large salbutamol Ventolin^TM^
Actuations of a first full pMDI		
Cartridge weight		
Full	15.64 g	28.75 g
Empty	7.76 g	11.43 g
Total volume	1640 ml	3885 ml^a^
Actuations of a second full pMDI		
151–200 (50)	350 ml	800 ml
201–210 (10)	70 ml	160 ml
211–220	70 ml (last “sensing”)	160 ml (last “sensing”)
221–230	65 ml	100 ml
231–240	30 ml	70 ml
241–250	5 ml	40 ml
251–260	0 ml	15 ml
Total volume	1640 ml	3745 ml^a^

^a^140 ml less total propellant gas volume of second large pMDI

Avogadro’s hypothesis theoretical gas volumes at 20 °C were: Small pMDI 1730 ml (measured 1640 ml): Large 4075 ml (measured 3885 ml).

### Falls in oxygen

Table 3—maximum fall after 10 puffs in the smallest spacer was from 21 to 11%.

**Table 3:** Valved holding chamber type; their volumes; and drop in oxygen percentage from 21% air per actuation

Spacer type	Volume (ml)	1 puff (O_2_ %)	2 puffs (O_2_ %)	10 puffs (O_2_ %)
Ablespacer™	126	19	17	11
Vortex™	180	19	18	13
BabyHaler™	393	20	19	16
Volumatic™	788	20	19	17

### Oxygen falls in ventilated circuit reservoir bag

Table 4 shows fall in oxygen from 100% after five actuations of HFA 134a and HFA 227ea propellant pMDIs. The results of ×5 actuations of HFA 227ea gas propelled Intal™ (total of 80 ml), delivered during in vitro ventilation into a 1 l bag should decrease oxygen percentage by 80/1000 × 100% = 8%, therefore 92% should be the theoretical percentage result, the range was 79–91%. Interference by HFA 227ea with the oxygen analyser, poor mixing in the reservoir bag, the research methods and execution may explain these results.

**Table 4:** Falls in percentage from 100% oxygen after five actuations of pMDIs into four ventilator delivery devices

	5× Duolin™ 45 ml (O_2_ %)	5× Respigen™ 60 ml (O_2_ %)	5× Vannair™ 45 ml (O_2_ %)	5× Intal™ 67.5 ml (O_2_ %)
L-Trace™	88	87	89	81
Spirale™	86	87	89	82
Intersurgical™	87	86	87	79
RT 200 (ventilator tubing)	90	91	93	91

## Discussion

No research has been performed to quantify the bronchodilating actions of HFA134a and 227ea. Delivery from pressurised metered dose inhalers (pMDIs) relies on these two fluorinated hydrocarbon propellants with low boiling points. The majority of pMDIs use hydrofluoroalkane (HFA) 134a, norflurane, formula CF_3_CFH_2_, an anaesthetic agent of intermediate potency, described in 1967. The gas was studied in dogs, cats and monkeys, and required 50% in oxygen to anaesthetise dogs: “Action was rapid and readily reversible, overdosage is difficult and vital functions appear to be protected even at very high concentrations”. No human studies were performed on anaesthetic effects. This publication is not cited in any toxicology study of the propellants, possibly because the Shulman and Sadove [12] publication title used the alternative name for HFA134a of 1,1,1,2-tetrafluoroethane (TFE). This has a similar chemical structure to the inhalational anaesthetic agent halothane; a bromine and chlorine is replaced by an additional fluorine and hydrogen. Halothane and all other hydrofluorocarbon (hydrofluoroalkane) anaesthetic agents are potent smooth muscle relaxants of gut, vasculature, uterus and bronchi. HFA 227ea, apaflurane, CF_3_CFHCF_3_, the other pMDI propellant, is chemically similar to the inhalational anaesthetic agent isoflurane but has no anaesthetic activity. Both propellants are refrigerants, the boiling point of HFA 134a is minus 26.3 °C, and of HFA227ea is minus 17.3 °C. Corticosteroid pMDIs contain 13% ethanol to improve solubility, one salbutamol pMDI (Respigen™, Mylan, Auckland, NZ) has 7% ethanol; all other salbutamol HFA MDIs have 1% ethanol added. The high percentage of ethanol is the likely reason for cough following inhalation of corticosteroid.

For 50 years in the United States of America, an over-the-counter-purchase adrenaline (epinephrine) CFC pMDI, delivered 220 mg of adrenaline per puff, and for solubility reasons contained 34% ethanol. A proposed new HFA134a pMDI delivering 125 mg of adrenaline with 1% ethanol was not passed for public use by the Federal Drugs Administration in 2014 [13].

The results of these simple easily repeatable experiments may have more educational and research use than clinical relevance, but demonstrate why it is correct for health carers, patients and parents to use multiple actuations of pMDIs through VHCs. Oxygen driven jet nebulisation of saline diluted bronchodilator drugs has limited pulmonary absorption, because water vapour carriage of drugs can only achieve a partial pressure of 47 mmHg (6.3 kPa) which is the pressure of saturated water vapour at 37 °C. This limits the amount of drug that can be delivered below the carina, unlike the gas HFA134a which produces a partial pressure related to the percentage in that breath, and hence carries particles of asthma drugs down to alveoli. Falls in oxygen levels because of high inhaled gas percentage displacing oxygen may have implications of hypoxia for recreational users of asthma inhalers, who are known as “huffers”. Purloined asthma inhalers, when actuated into balloons, empty plastic drink bottles, or other vessels, give a “high” to the person inhaling, and the inhaled gas will have a low oxygen content [14, 15]. In CFC propellant metered dose inhalers it was thought that salbutamol rather than the propellant caused a “high”, because there were no cases of cortico steroid inhaler abuse [16, 17]; an additional anaesthetic or stupifiant effect is likely with current HFA134a pMDIs.

Patients or doctors who use multiple actuations of pMDIs at one time into VHCs, spacers, and ventilator delivery devices in acute severe and life-threatening asthma, or for reversal of bronchospasm in anaphylaxis, may be affected by a reduction of oxygen and a slight sedative effect of HFA134a. HFA 134a (also known as R134a) is used as an automobile air conditioner refrigerant, but because it has a global warming potential (GWP) of 1410, is likely to be withdrawn from use, as only compounds with a GWP of less than 150 will be allowed. GWP is a 100 year warming potential of 1 kg of a gas relative to 1 kg of carbon dioxide, which has a GWP of 1. In pharmaceutical factories, HFA134a and HFA227ea, and asthma medication with added ethanol to aid solubility, are introduced into different sized drawn aluminium bottles by a “cold transfer method” at minus 55 °C (Information from manufacturers of Airomir^R^, 3 M Health Care Ltd), the propellant is a liquid with a saturated vapour above.

A toxicology study looked at eight volunteers, four male, four female, who inspired pure HFA 134a, HFA 227ea and CFC in air when inside a whole body exposure chamber, up to a maximum of 8000 parts per million (0.8%) for HFAs, and 4000 ppm CFC for 1 h on eight separate occasions. Why an 8000 ppm concentration was chosen is not explained. EKG (ECG), blood pressure, pulse, and lung function by peak expiratory flow rate (PEFR), measured 75 min after cessation of exposure, and serial blood samples were taken. There were no statistical changes in clinical evaluation parameters [6]. However, after 75 min, a bronchodilating effect of HFA 134a on normal tone bronchial muscle which may change PEFR, would be long-gone.

In the introduction the authors of this study mention unpublished data suggesting that; “The threshold for cardiac sensitisation in dogs was 75,000 ppm (7.5%) for HFA 134a and 100,000 ppm (10%) for HFA 227ea”, but do not explain what they mean by cardiac sensitisation or describe the delivery gas which may have been air. They do not reference Shulman and Sadove’s publication which stated that at 50–80% of TFE inhalational agent (HFA 134a) delivered in oxygen to dogs; “The electrocardiograph is usually quite stable, with either a sinus or a nodal rhythm”. Exposure of rats up to 50,000 ppm (5%) HFA 134a 6 h per day, 5 days a week for 2 years produced an increase in Leydig cell tumours, common in rats, but not humans.

Another toxicology study gave pure HFA 134a; HFA 134a with salbutamol; and pure CFC metered dose inhalers to twelve healthy male volunteers of up to 16 inhalations from pMDIs [5]. Pulmonary function, (FEV_1_, FEF_25–75%_), cardiovascular performance, (heart rate and blood pressure) were measured after each incremental dose. HFA 134a-salbutamol produced statistically significant dose-related increases in heart rate, systolic blood pressure and tremor and a significant dose—related decrease in serum potassium. A spirometric respiratory function test was performed 20 min after the last of the 16 MDI doses, and although HFA 134a salbutamol statistically improved FEV_1_ and FEF_25–75%_, an opportunity to see if there was a bronchodilating effect of HFA 134a alone may have been lost because of this delay and a consequent wash out of the propellant; no statistical change was seen. A further study exposed five subjects to pure HFA 134a in air via a one way face mask (the manufacturer of the mask was not described) to see what happened on exposure if HFA 134a was used as a flame suppressant. Subject #3 at 4000 ppm (0.4%) exhibited a rapid drop in pulse and blood pressure and fainted, and subject #5 exhibited an increase in blood pressure and heart rate about 10 min after initiation of the exposure. Subject #1 was exposed to HFA 227ea at 6400 ppm (0.64%) for 3 min and, quote; “pulse rose rapidly and uncontrollably to double the baseline (pre-exposure) value. The exposure was terminated after 3.5 min. The subject’s pulse returned to its pre-exposure level within 30 s after exposure was terminated”. A day later the breathing system without HFA227ea was used by the same subject with no untoward effect. No further exposures were attempted [18]. Malignant hyperpyrexia (MH) is triggered in a dose related fashion by inhalational anaesthetic agents in susceptible individuals; research is required to see if HFA134a and HFA227ea have this undesirable property. R134a, the refrigerant in automobile air conditioning units is available for study. The single volunteer subject #5, breathing HFA134a, as previously mentioned, who had an increase in blood pressure and a tachycardia, lead to the experiments being abandoned.

A reduction in oxygen concentration in spacers causing mixtures low in oxygen to be inhaled should have little untoward consequence. A benefit of bronchodilation and a subsequent breath of air containing 21% oxygen, should counter a breath of low inhaled oxygen. If spacer inhalation therapy interrupts continuous oxygen delivery via a face mask during a severe asthma attack, this reduces the danger of a hypoxic inhaled mixture. A child with a 1600 ml lung volume inhaling 10 × 16 ml from a large salbutamol inhaler will have a maximum  10% of HFA134a in that breath which is meant to be held as long as possible. After this breath hold the spacer may be empty of gas and drug, so further deep breaths from this spacer are pointless. The time for gas to escape from a VHC has not been determined.

“Asthma spacers used with MDIs remove the need for coordination between actuation and inhalation, reduce the velocity of the aerosol and allow time for evaporation (and warming) of the propellant so that a larger proportion of the particles can be inhaled and deposited in the lungs” [11]. After activation of the metered dose inhaler, the nomenclature of the product is variously described in literature as an “aerosol”, “vapour”, or “gas”, at that particular temperature; HFA134a and HFA227ea act as gases in the lung.

A large difference in lung deposition of corticosteroid carried by HFA 134a and CFC propellant has been seen in nine healthy volunteers breathing either HFA134a or chlorofluorocarbon (CFC) propellant carrying radio-active labelled beclometasone. 53% with HFA and 4% CFC lung deposition occurred. A smaller particle size of HFA134a beclometasone of 1.1 μ to CFC beclometasone of 3.5 μ was thought to account for the difference. Particle size distribution from the pMDIs was determined by an Andersen 1 ACFM Particle Sizing Sampler (Mark 11; Andersen Samplers; Atlanta, GA) and a Quartz Crystal Microbalance Cascade Impactor System (California Measurements; Sierra Madre, CA) [19]. A corticosteroid “carrier” effect of HFA 134a as a vapour or gas, and a reduction in resting bronchial tone because of a bronchial smooth muscle relaxing effect is another reason for better lung distribution. Particle or droplet size is measured in vitro, but in the lungs, droplets must evaporate before absorption, and particles can descend further within a partial pressure generating gas.

## Conclusion

A greater volume of HFA134a propellant per actuation is produced by large salbutamol metered dose inhalers, 16 ml versus 7 ml for the smaller. If this propellant on its own relaxes bronchial smooth muscle and the gas aids drug delivery (including corticosteroid), then large metered dose inhaler actuation gives maximum bronchodilation for patients and athletes (and huffers). Exhaustion of pMDIs can be sensed by shaking, inhalers are now produced using a mechanical system with a numbered dial to count down each actuation. Further research on bronchial smooth muscle effects of HFAs may be performed with available propellant—only pressurised metered dose inhalers which are used to assess efficiency of inhalation by patients.

## Abbreviations

HFA: hydrofluoroalkane
CFC: chlorofluorocarbon
pMDI: pressurised metered dose inhaler
VHC: valved holding chamber
GWP: global warming potential
